# Frequency of obesity and metabolic syndrome in childhood leukemia and lymphoma survivors

**DOI:** 10.1186/s13098-022-00790-4

**Published:** 2022-01-24

**Authors:** İbrahim Kartal, Abdurrahman Alaçam, Ayhan Dağdemir, Cengiz Kara, Oğuz Salih Dinçer, Canan Albayrak, Murat Elli

**Affiliations:** 1 Division of Pediatric Hematology and Oncology, Department of Pediatrics, Faculty of Medicine, Ondokuz Mayıs University, Samsun, Turkey; 2 Department of Pediatrics, Faculty of Medicine, Ondokuz Mayıs University, Samsun, Turkey; 3 Division of Pediatric Endocrinology, Department of Pediatrics, Faculty of Medicine, Istinye University, İstanbul, Turkey; 4 Division of Pediatric Hematology and Oncology, Department of Pediatrics, Faculty of Medicine, Medipol University Hospital, İstanbul, Turkey

**Keywords:** Childhood leukemia-lymphoma, Obesity, Metabolic syndrome, Cancer survivor

## Abstract

**Objectives:**

In this study, it was aimed to determine the prevalence and clinical features of obesity and metabolic syndrome, which are long-term effects of survivors after treatment in children with leukemia and lymphoma.

**Patients and Methods:**

Patients with leukemia and lymphoma, who were diagnosed between 2000 and 2012 (at least 2 two years after remission) were included. Data obtained through reviewing the family history, demographic characteristics, anthropometric measurements, and laboratory parameters (blood glucose, lipid, and insulin levels) were analyzed and compared at the time of diagnosis, after the treatment and at time of the study.

**Results:**

Eighty nine patients (45 boys, 44 girls) were included (mean age: 14.7 ± 4.3 years): 77.5% had acute lymphoblastic leukemia, 11.2% had acute myeloid leukemia, and 11.2% had lymphoma. Overall, 46% patients had received radiotherapy, 7% had undergone surgery, and 2.2% had received stem cell transplantation in addition to chemotherapy. The mean duration of treatment was 2.4 years, and the time elapsed after treatment was 4.9 years. While only one had obesity at the diagnosis, a significant increase in obesity (20%), hypertension (15.7%), hyperglycemia (15%), insulin resistance (35%) were observed at the time of study, and family history of hypertension, dyslipidemia, and cardiovascular diseases were significantly higher in this subgroup.

**Conclusion:**

The prevalence of metabolic syndorme is higher in children with leukemia and lymphoma after treatment, and begins to increase with the initiation of treatment and continues to increase over time. These children should be followed-up for late-effects including metabolic syndrome through life-long period.

## Introduction

A large proportion of patients (approximately 60%) who are long-term survivors of childhood cancer experience complications or “late effects” that reduce their survival chances or quality of life later in their lives as a result of long-term effects of cancer treatments [1]. Significant advances have been made in the survival rates of patients with leukemia and lymphoma; particularly in the last few decades, 5-year survival rates have reached 89% for acute lymphoblastic leukemia (ALL), 61% for acute myeloid leukemia (AML), 96% for Hodgkin lymphoma, and 89% for non-Hodgkin lymphoma [2]. This has led to the necessity of careful evaluation of long-term morbidity and mortality outcomes after treatments that provide a high survival advantage. The well-known long-term side effects of cancer treatments include hypertension (HT), dyslipidemia, impaired glucose metabolism, and obesity [3–6].

Obesity is an abnormal or excessive accumulation of body fat, defined by the World Health Organization as a body mass index (BMI) of ≥ 30 kg of body weight per square meter of height (kg/m^2^). The reported prevalence of obesity among adult survivors of childhood ALL ranges from 11 to 56% and varies based on reporting method and cohort characteristics [7].

The survivors of hematological malignancies are predisposed to the development of metabolic syndrome (MetS), which is a clinical picture formed by the combination of interrelated risk factors such as high blood pressure [≥ 130/85 mm of mercury (mmHg)], high waist circumference(> 102 cm (cm) in males and > 88 cm in females), blood lipid disorders (triglyceride levels > 150 mg/dl, high density lipoprotein cholesterol (HDL-C) < 40 mg/dl), and impaired glucose tolerance (glucose ≥ 100 mg/dl), which in turn increases the risk of cardiovascular disease (CVD) and type 2 diabetes [8, 9]. Particularly, cardiovascular morbidity and mortality are noted to increase in cancer survivors [10].

In this study, we aimed to determine and present the prevalence of obesity and MetS in children with leukemia and lymphoma in post-treatment remission.

## Patients and methods

### Study group

Patients aged ≥ 6 who were diagnosed with leukemia and lymphoma in Ondokuz Mayıs University, Faculty of Medicine, Department of Pediatrcis, Division of Pediatric Hematology and Oncology, located in the northern coast of Turkey, between 2000 and 2012 and survived at least 2 years after completing all their treatments were included in the study. The study was approved by the Ondokuz Mayıs University Clinical Research Ethics Committee.

### Methods

In addition to the demographic data of the patients, family history, physical activities, sleep time, time spent in front of the screen, place of residence, family structure, and economic status were obtained. Anthropometric measurements and laboratory parameters (glucose, Total, HDL and LDL, cholesterol, Triglyceride levels, insulin level) were reviewed. Subgroups of the patients with and without obesity and MetS were compared with respect to these parameters.

### Data collection

Demographic data were obtained from hospital records.

### Anthropometric measurements

Bodyweight (BW) and height measurements of the patients were obtained and the body mass index (BMI) values were calculated at the diagnosis, at the end of treatment, and during the study at the last visit. These parameters were evaluated using the Turkish children percentile chart published by Neyzi et al. [11], and z-score (SDS) values for BW, height, and BMI were calculated. The changes in BMI was revealed by calculating the differences among SDS values of BMI at the three steps of the study mentioned above. We used the parameters of the percentile values of waist circumference by Hatipoğlu et al., which reported for Turkish children aged between 7 and 17 years, to determine the percentile values of the waist circumference [12].

### Laboratory methods

Serum glucose, insulin, triglyceride, and high-density lipoprotein (HDL) and LDL, cholesterol levels were tested on the same day using blood samples collected after 8–12 h of overnight fasting. Insulin resistance was evaluated using Homeostasis Model Assessment of Insulin Resistance [HOMA-IR; fasting insulin (μU/ml) × fasting blood glucose (mg/dl)/405]. A HOMA index of ≥ 2.5 indicated insulin resistance.

### Definition of obesity and MetS

Obesity was defined as a BMI of ≥ 95th percentile in children and adolescents and BMI of ≥ 30 kg/m^2^ in those aged 18 years and above. Those with a BMI between the 85th and 95th percentile were considered overweight. Based on the measurements of waist circumference of Turkish children by age, those with a BMI of > 90th percentile were considered to have abdominal obesity.

MetS diagnostic criteria for children and adolescents published by the International Diabetes Federation (IDF) in 2005 were used to determine MetS [13]. According to IDF, patients were categorized into the three following groups based on age: 6 to < 10, 10 to < 16, and > 16 years. Central obesity was the main criteria for all age groups. Between the ages of 6 and 10, waist circumference above the 90th percentile and family history were enough for MetS with no need for biochemical features. For those aged > 10 years, two of the four criteria were sought in addition to obesity. The presence of HT was comfirmed if the patient receiving antihypertensive medication or having measurements higher than the 130/85 mmHg by IDF. HT in children aged < 10 was defined as being above the 95th percentile according to the Turkish childhood percentile values determined by Tümer et al. [14].

### Statistical analysis

The descriptive statistics were expressed as frequency distributions and percentages for categorical data and mean and standard deviation or median and minimum–maximum values for numerical data. Chi-square statistics were used to compare categorical data and Mann–Whitney U statistics were used to compare numerical data between independent data groups of the study. All analyses were evaluated based on two-tailed hypothesis test with a 5% probability for making a type-I error. Statistical analyses were performed using SPSS software version 21 (IBM Corp., Armonk, NY, USA).

## Results

Eigthy nine patients were eligibile for the study: 69 acute lymphoblatic leukemia, 10 acute meyloblastic leukemia, 10 lymphoma. Boys were 50.6% and girls were 49.4% of them, and the median and mean ages were 15.3 (7–22.3) and 14.7 ± 4.3 years, respectively (Table 1). Patients aged ≥ 16 years constituted the largest age group, including 44.9% patients. In terms of birth weight, 79.8% patients were appropriate for gestational age.

**Table 1:** Anthropometric measurements of the patients

	Entire group (n = 89)	Male (n = 45)	Female (n = 44)	p-value
Body weight				
Kilograms, Mean ± SD	56 ± 19.1	61.5 ± 19.9	50.3 ± 16.6	
Percentile, Median (min–max)	62 (1–100)	64 (3–100)	59 (1–100)	0.525
SD score, Median (min–max)	0.4 (− 2.2–4)	0.4 (− 1.8–3.3)	0.4 (− 2.2–4)	0.608
Height				
Centimeters, Mean ± SD	155.4 ± 16.9	160 ± 18	150.7 ± 14.5	
Percentile, Median (min–max)	50 (1–97)	46 (2–97)	52 (1–84)	0.977
SD score, Median (min–max)	0 (− 2.6–2)	− 0.1 (− 2.1–2)	0.1 (− 2.6–1.2)	0.938
Body Mass Index				
kg/m^2^ Mean ± SD	22.4 ± 4.6	23.4 ± 4.5	21.5 ± 4.5	
Percentile, Median (min–max)	67 (3–100)	66 (4–100)	69 (3–100)	0.329
SD score, Median (min–max)	0.4 (− 1.9–3.4)	0.4 (− 1.8–2.9)	0.5 (− 1.9–3.4)	0.337
BMI classification, n (%)				
Obese	18 (20.2)	11 (22.4)	7 (15.9)	0.814
Overweight	12 (13.5)	6 (13.3)	6 (13.6)	
Normal weight	57 (64)	27 (60)	30 (68.2)	
Underweight	2 (2.2)	1 (2.2)	1 (2.3)	
Waist circumference, Mean ± SD				
Centimeters, Mean ± SD	74.2 ± 12.5	78.7 ± 12	69.6 ± 11.5	
Percentile, median (min–max)	74.9 (60–94)	81.1 (60–94)	73.7 (60.1–81.1)	< 0.001
Waist circumference classification, n (%)				
Normal	57 (64)	26 (57.8)	31 (70.5)	0.213
Obese	32 (36)	19 (42.2)	13 (29.5)	
Blood pressure, mean ± SD				
Systolic	111.4 ± 15.4	115.4 ± 15.4	107.2 ± 14.4	0.013
Diastolic	68.9 ± 10	70.7 ± 10.6	67.1 ± 9	0.150
Blood pressure classification, n (%)				
Normotensive	75 (84.3)	34 (75.6)	41 (93.2)	0.022
Hypertensive	14(15.7)	11 (24.4)	3 (6.8)	

*SD* standard deviation, *BMI* body mass index

Regarding diagnostic characteristics of the patients, it was found that 77.5% had ALL, 11.2% had AML, and 11.2% had lymphoma; the mean age at diagnosis was 7.4 ± 4.3 years, and BMI at diagnosis was 17 ± 2.4 kg/m^2^. Based on these BMI values, 88.8% patients were classified as having a normal weight.

When the treatment characteristics and end-of-treatment measurements were examined, it was seen that 46.1% patients received radiotherapy (RT), 6.7% underwent a surgical procedure, and 2.2% underwent stem cell transplantation. The mean duration of treatment was 2.4 ± 0.8 years, and BMI at the end of treatment was 18.6 ± 3.1 kg/m2. According to these BMI values, 13.5% patients were obese and 11.2% were overweight. Furthermore, the mean elapsed time after treatment was 4.9 ± 2.9 years.

**Fig. 1:**
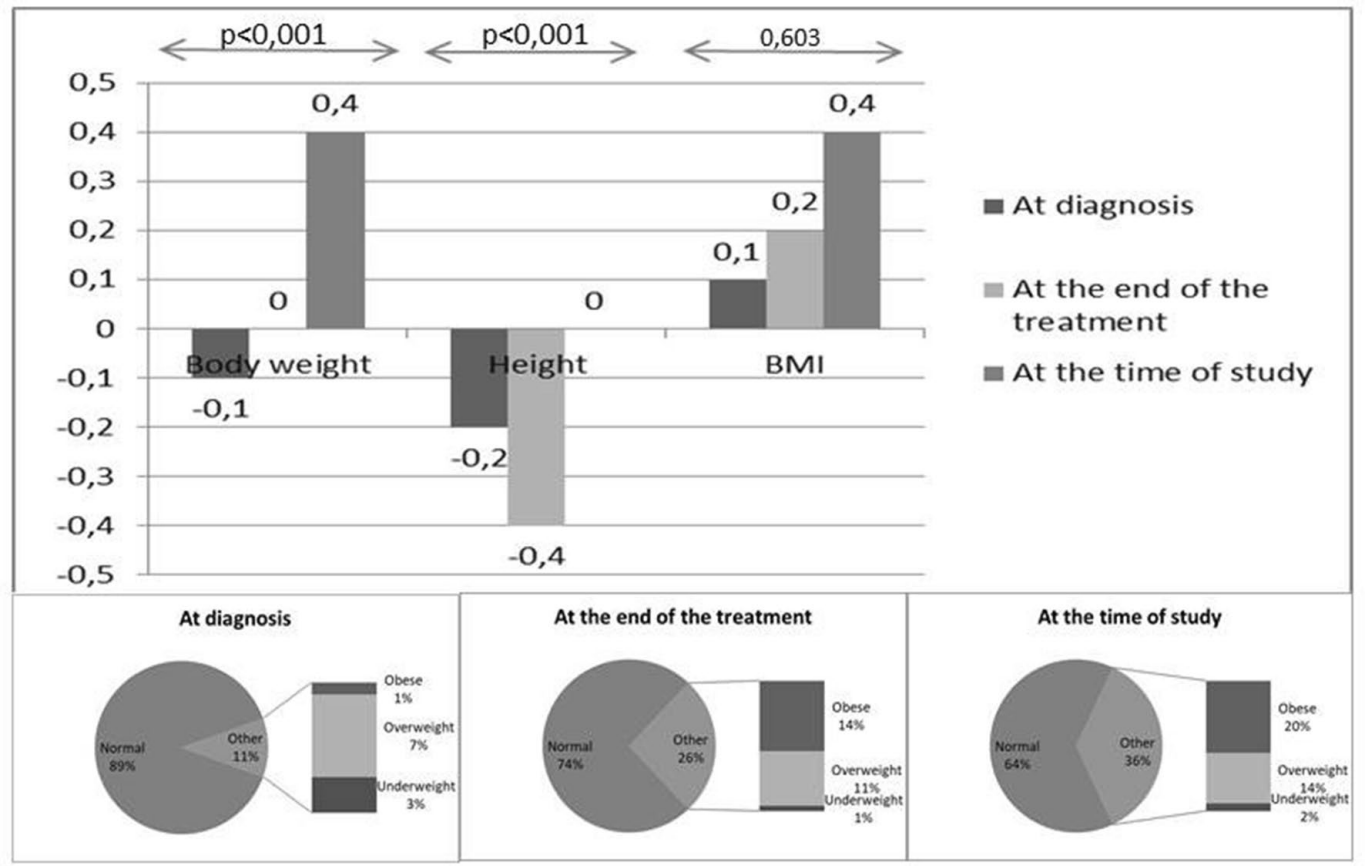
Variation in anthropometric measurements over time

The changes in anthropometric features measured at diagnosis, at the end of treatment, and during the study are summarized in Table 2. All anthropometric measurements showed a significant increase over time, and the obesity rates of the patients also increased over time (Fig. 1).

**Table 2:** Variation in anthropometric measurements over time

	At diagnosis	At the end of the treatment	At the time of study	p­-value
Body weight				
Kilograms, Mean ± SD	27.3 ± 14.8	39.4 ± 16.1	56 ± 19.1	
Percentile, Median (min–max)	48 (1–95)	46 (1–136)	62 (1–100)	< 0.001
SD score, Median (min–max)	− 0.1 (− 2.4–1.6)	− 0.04 (− 2.4–2.5)	0.4 (− 2.2–4)	< 0.001
Height				
Centimeters, Mean ± SD	121.6 ± 24.6	133 ± 22.1	155.4 ± 16.9	
Percentile, Median (min–max)	42 (2–99)	33 (1–99)	50 (1–97)	< 0.001
SD score, Median (min–max)	− 0.2 (− 2–2.3)	− 0.44 (− 2.6–2.5)	0 (− 2.6–2)	< 0.001
Body mass index				
kg/m^2^, Mean ± SD	17 ± 2.4	18.6 ± 3.1	22.4 ± 4.6	
Percentile, Median (min–max)	48 (1–96)	56 (1–100)	67 (3–100)	< 0.001
SD score, Median (min–max)	0.1 (− 5.2–3.5)	0.18 (− 2.5–2.9)	0.4 (− 1.9–3.4)	0.603
BMI classification, n (%)				
Obese	1 (1.1)	12 (13.5)	18 (20.2)	< 0.001
Overweight	6 (6.7)	10 (11.2)	12 (13.5)	
Normal weight	79 (88.8)	66 (74.2)	57 (64)	
Underweight	3 (3.4)	1 (1.1)	2 (2.2)	

*SD* standard deviation, *BMI* body mass index

There was no significant difference between sexes in terms of BW SDS, height SDS, BMI SDS, and waist circumference percentile values; however, high systolic blood pressure were found to be significantly higher in boys (p = 0.013). When the BMI categories (obese + overweight and normal + underweight) were compared in terms of sex, no significant difference was observed (p = 0.411).

When the lifestyle and social characteristics of the patients were evaluated, it was found that 68.5% spent > 3 h in front of a screen, 55.1% had physical activity within normal limits, 56.2% slept between 6 and 9 h, 86.5% had a nuclear family structure, 52.8% had an income of 1300–4000 TL (350–1100$), and 73% lived in urban areas. When the lifestyle and social characteristics of the groups compared according to the presence of MetS, patients with MetS performed less physical activity (p = 0.023).

On evaluating the family histories of patients, it was found that 31.5% of patients had a family history of HT, 14.6% had a family history of diabetes mellitus (DM), 60.7% had a family history of obesity, and 22.5% had a sibling with obesity; moreover, 20.2% and 25.8% patients had a family history of dyslipidemia and CVD, respectively.

When family histories were evaluated, it was observed that the presence of HT (p < 0.001), dyslipidemia (p = 0.003), and CVD (p < 0.001) in the family was significantly high in patients with MetS.

**Fig. 2:**
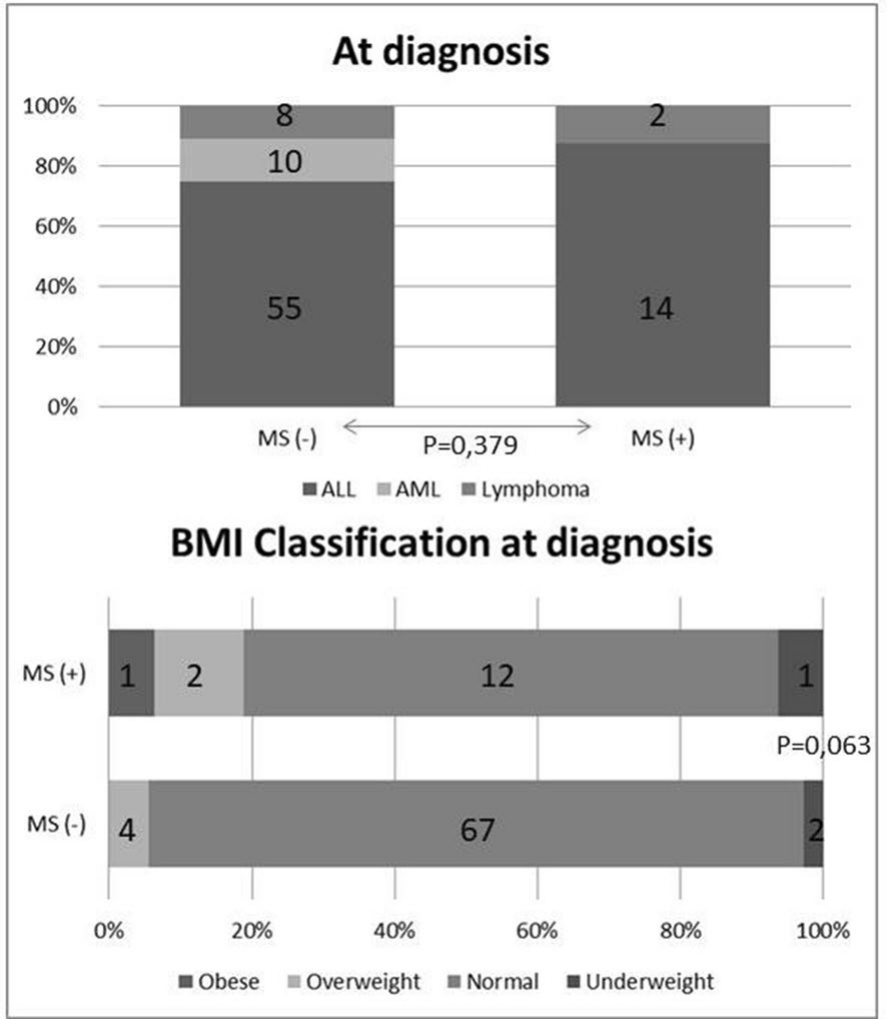
Diagnostic features according to the presence of metabolic syndrome

The diagnostic features of patients with MetS are summarized in Table 3. Weight percentile (p = 0.013) and weight percentile SD (p = 0.017) values at diagnosis and BMI percentile (p = 0.011) and SD (p = 0.046) values at diagnosis were significantly high in patients with MetS (Fig. 2). The time elapsed after treatment (mean ± SD) of the groups of those with MetS or not were 4.9 ± 2.9 and 4.9 ± 3.0 years, respectively, (p 0.894).

**Table 3:** Diagnostic features of patients with and those without metabolic syndrome

	MS (−) (n = 73)	MS ( +) (n = 16)	p-value
Diagnosis, n (%)			
ALL	55 (75.3)	13 (87.5)	0.379
AML	10 (13.7)	0 (0)	
Lymphoma	8 (11)	2 (12.5)	
Age at diagnosis, Mean ± SD	7.8 ± 4.4	5.7 ± 3.4	0.097
Weight at diagnosis			
Kilograms, Mean ± SD	28.5 ± 15.6	22 ± 8.6	
Percentile, Median (min–max)	44 (1–92)	64 (1–95)	0.013
SD score, Median (min–max)	− 0.2 (− 2.4–1.4)	0.3(− 2.2–1.6)	0.017
Height at diagnosis, Mean ± SD			
Centimeters, Mean ± SD	123.4 ± 25.6	113.3 ± 18.5	
Percentile, Median (min–max)	42 (4–95)	45 (2–99)	0.532
SD score, Median (min–max)	− 0.2 (− 1.8–1.6)	0.1 (− 2–2.3)	0.326
BMI at diagnosis, Mean ± SD			
BMI, Mean ± SD	17.1 ± 2.6	16.6 ± 1.2	
Percentile, Median (min–max)	46 (1–89)	65.5 (5–96)	0.011
SD score, Median (min–max)	0 (− 5.2–3.5)	0.4 (− 1.7–1.8)	0.046
BMI classification at diagnosis, n (%)			
Obese	0 (0)	1 (6.3)	0.063
Overweight	4 (5.5)	2 (12.5)	
Normal weight	67 (91.8)	12 (75)	
Underweight	2 (2.7)	1 (6.3)	

*MS* Metabolic syndrome, *SD standard deviation*

The retrospective evaluation of the groups at the diagnsosis revealed no significant difference of the obese + overweight and normal + underweight groups categorized according to the BMI at diagnosis revealed that the rates of insulin resistance (p = 0.717), obesity (p = 0.167), and MetS (p = 0.107) (Table 4).

**Table 4:** Insulin resistance, obesity and metabolic syndrome rates according to BMI at diagnosis

	BMI at the time of diagnosis		
	Obese + Overweightn (%)	Normal + Low Weight n (%)	p-value
Insulin resistance	2 (28, 6)	29 (35, 4)	0.717
Obesity	1 (14, 3)	17 (20, 7)	0.167
Metabolic syndrome	3 (42, 9)	13 (15, 9)	0.107

According to the results of biochemical evaluations, it was determined that 14.6% patients had hyperglycemia, 31.5% had low HDL, 11.2% had high triglyceride levels, and 34.8% had insulin resistance at the diagnosis and there was no high total cholesterol levels that could effect the cardivascular risk.

When patients were analyzed in terms of risk factors included in the definition of MetS (i.e., abdominal obesity, HT, low HDL, high triglycerides, and fasting hyperglycemia), at least one risk factor was present in 60 (67.4%) patients, at least two in 24 (26.9%), at least three in 12 (13.4%), and four in one (1.1%) patient.

## Discussion

Childhood cancer continues to be an important health concern. Owing to the improvements in chemotherapy and supportive treatments, the survival rates in childhood cancers have increased and the long-term survival rates are gradually increasing [2]. Therefore, the long-term morbidity and mortality outcomes of treatments that provide a survival advantage need to be carefully evaluated. The frightening increase in childhood obesity in recent years has resulted in increased prevalence of MetS, a significant problem, particularly in adolescents. It was revealed that obesity, HT, hyperlipidemia, DM, and consequently, MetS and CVDs are seen more frequently in patients who have been treated for leukemia and lymphoma those in general population. Moreover, they carry the risk of being diagnosed with MetS and CVDs at an earlier age [7].

Although there are numerous other studies that describe weight gain during ALL therapy as well as the presence of obesity in ALL survivors, recent studies show that weight gain during induction as a potential predictor for later obesity, most likely due to glucocorticoids [15]. Long-term use of corticosteroids in lymphoreticular malignancies, unlike solid tumors, must be the major factor for obesity in children, but we were not able to compare whether corticosteroids or any other drugs to cause obesity and MetS in our children because all them received the same drugs in this study.

Because obesity is a multifactorial disease affected by genetic, behavioral, environmental, and cultural factors, its prevalence is expected to be different in studies from different regions. In 1975, the prevalence of overweightedness and obesity in most European countries was < 10% and < 5%, respectively, whereas in 2016, this trend had reversed, showing an alarming increase in the number of European countries with an increased prevalence of overweightedness (> 30%) and obesity (> 10%) [16]. In the USA, the National Health and Nutrition Examination Survey reported that 16.3% of children and adolescents are obese [17]. In Turkey, the rate of obesity in children varies between 6 and 10% and the rate of overweight children varies between 8 and 15% [18, 19]. In a study conducted by screening a total of 4120 secondary and high school students in Samsun, the region in which our hospital provides healthcare services, the prevalence of obesity was found to be 7.3% in girls and 4% in boys, with an overall prevalence rate of 5.5% [20].

In this study, obesity and overweightedness were detected in 1 (1.1%) and 6 (6.7%) cases at the time of diagnosis and in 12 (13.5%) and 10 (11.2%) cases at the end of treatment, respectively. On the last visit, the number of obese and overweight patients had increased to 18 (20.2%) and 12 (13.5%), respectively. This dramatic increase in obesity and overweightedness was also statistically significant (p < 0.001).

When the patients were examined during the study, a total of 30 overweight cases (33.7%) were detected; 18 (20.2%) cases were obese; and 12 (13.5%) cases were overweight. Compared to the data of Turkey, the rate of overweightedness in our patients was similar to that in the normal population, while the rate of obesity in our patients was significantly higher than that in the normal population. This result, which is consistent with the literature, shows an increased prevalence of obesity in patients who are treated for cancer in childhood compared with that in the healthy population.

In this study, there were 16 (18%) cases that met the criteria of MetS. The prevalence of MetS was found to be higher than that of the general population and similar to the rates observed in different studies conducted with patients who received cancer treatment. When the patients were analyzed in terms of risk factors included in the definition of MetS (i.e., abdominal obesity, HT, low HDL, high triglycerides, and fasting hyperglycemia), at least one risk factor was present in 60 (67.4%) patients, at least two in 24 (26.9%), and at least three in 12 (13.4%) patients. Although there are many studies and prevalence data on obesity and MetS in individuals treated for childhood cancers, the underlying pathophysiological processes and mechanisms contributing to the increased CVD risk are still unknown, and the etiology is thought to be multifactorial. Potential mechanisms for the increased risk of MetS include disruptions in leptin and adiponectin levels, early adiposity rebound, pancreatic insufficiency, poor dietary habits, sedentary lifestyle, and changes in the gut microbiome composition [21–23]. Frequent absenteeism from school during treatment increase inactivity. During treatment, parents tend to be overprotective, limit outdoor activities, and overfeed their children [24]. The reasons that decrease physical activity in children with cancer are general malaise, muscle weakness, osteopenia, decreased lung functions, and cardiomyopathy in addition to low self-confidence [25].

Corticosteroids and asparaginase used in the treatment of leukemia and lymphoma are thought to have effects on glucose metabolism [26]. Insulin resistance was detected in 31 (34.8%) of the 89 patients in our study, whereas impaired fasting glucose was detected in 13 (14.6%) patients. Nine (10.1%) patients had both insulin resistance and impaired fasting glucose. Consistent with the literature, the prevalence of insulin resistance and impaired fasting glucose was found to be high in patients who had leukemia and lymphoma treatment [27]. Among the patients with insulin resistance, 45.2% were obese (n = 14), 9.7% were overweight (n = 3), 41.9% had normal weight (n = 13), and 3.2% were underweight (n = 1). Although insulin resistance was more common in the obese group, the difference was not statistically significant. Insulin resistance was observed in 17 (39.5%) patients aged < 6 years and 14 (34.5%) patients aged ≥ 6 years at the time of diagnosis. Although a higher rate of insulin resistance was observed in those aged < 6 at the time of diagnosis, the difference between the two age groups was not statistically significant.

It is not fully known why blood pressure increases in post-treatment cancer survivors [28]. It has been suggested that endothelin dysfunction plays an important role in the development of CVDs [29]. Chemotherapy agents, such as alkylating agents, and RT damage vascular structures [30]. In studies conducted among the survivors of childhood ALL, the prevalence of HT (13–46.4%) was reported to be higher than that in the normal population [31–35]. However, in a few studies, none of the survivors had HT [36, 37]. In this study, HT was detected in 13 (14.6%) patients, and a higher rate of HT was observed in boy patients. The high frequency of HT in patients with leukemia and lymphoma indicates that blood pressure measurement should be a part of the routine outpatient examination in these children.

In the literature, it has been stated that receiving leukemia and lymphoma treatment at an earlier age (especially at < 5 years of age) is a risk factor for the development of late effects [38, 39]. It is known that early adiposity rebound in childhood is associated with an increased risk of obesity in adulthood. Adiposity rebound usually occurs between the ages of 5 and 7 years. Because ALL is frequently noted between the ages of 3 and 5 years, chemotherapy and RT may cause early adiposity rebound in these cases and may be the cause of obesity [40–43]. In the study by Razzouk et al., it was determined that age of < 6 years at the time of diagnosis is a risk factor for being overweight/obese in adulthood [44]. In a study conducted on ALL survivors in İzmir, the rate of obesity was higher in those diagnosed at the age of < 6 years than in those diagnosed at the age of > 6 years, and this difference was statistically significant [45]. In this study, although obesity and MetS rates were higher in those aged < 6 years and diagnosed with leukemia, this difference was not statistically significant. In addition, although the mean age at diagnosis of patients with MetS was lower than that of patients without MetS, this difference was not statistically significant.

Recently, some studies have stated that being overweight/obese at the time of diagnosis is the most important risk factor for the development of overweightedness/obesity in patients with cancer rather than other defined risk factors. This suggests that possible genetic and familial factors that may lead to obesity in patients are more prominent [44, 46, 47]. In our study, only one had obesity at the time of diagnosis who also obese at the last visit, but it was found that 31.5% had a family history of HT, 14.6% of DM, 60.7% of obesity, 25.8% of CVD, and 20.2% of dyslipidemia and 22.5% had a sibling with obesity. In the subgroup of MetS, the presence of HT (p < 0.001), dyslipidemia (p = 0.003), and CVD in the family was found to be significantly high (p < 0.001) and identified as a positive risk factor for the development of this syndrome. The fact of the higher prevalence of MetS in this study than those in healthy population of our region may suggest that MetS is primarily affected by environmental (chemoradiotherapy) and nutritional habits rather than familial factors, such as genetics. However, studies with larger groups can be conducted to obtain more definitive results.

Studies emphasize that nutritional habits and socioeconomic level of the family contribute toward developing childhood obesity [47, 48]. When the data in our study were examined, 16% patients were determined to have low income, 52.8% had moderate-income, and 29% had high income; additionally, 86.5% patients had a nuclear family structure and 73% lived in urban areas, and no significant difference was found between the group with MetS and that without MetS in terms of socioeconomic data.

Our patients have already been in childhood period and the increased risk factors for CVD could be expected over time.This fact requires regular follow-up of these patients in terms of the risk factors for CVD. Considering that most risk factors can be modified with lifestyle changes and medical interventions, cancer survivors should be encouraged to engage in preventive health care and healthy behaviors. Periodic follow-up of patients allows early detection and treatment of the risk factors that lead to the development of MetS and enables us to provide preventive suggestions such as proper nutrition and exercise, which aim to prevent a sedentary lifestyle that plays a role in the development of obesity. The probability of the follow-up of childhood cancer survivors in a cancer center or by an oncologist decreases in over time [49]. Therefore, it is important for family physicians, pediatricians, and internists to be aware of the risks in this population.

The lock of LDL-c measurements, family genetic and omic analysis due to technical problems and limited opportunities were among the limitations of the study. It was not planned as a randomized prospective study, and did not include large groups because of it was done in only one center.

## Conclusion

We found an increased frequency of obesity and MetS in children who received chemotherapy and other debilitating treatments such as radiotherapy for leukemia and lymphoma when compared to the baseline values, and we conclude that they become more prone to obesity and MetS as compared to the literature in general population. Emergency preventive measures are required in these lucky children who survived of cancer, so we can prevent some risks of serious health problems in later of life. Further prospective studies including control groups as well as in larger patients and more long-term follow-up periods are needed.

## Data Availability

The datasets used and/or analysed during the current study are available from the corresponding author on reasonable request.

## Abbreviations

SD: Standart deviation
BMI: Body mass index
MetS: Metabolic syndrome
